# Root Canal Shaping by Single-File Systems and Rotary Instruments: a Laboratory Study 

**Published:** 2015-03-18

**Authors:** Khaly Bane, Babacar Faye, Mouhamed Sarr, Seydina O Niang, Diouma Ndiaye, Pierre Machtou

**Affiliations:** a *Conservative Dentistry and Endodontics Service, Department of Dentistry, Faculty of Medicine, Pharmacy and Odontology-Stomatology, Cheikh Anta Diop University of Dakar, Dakar, Senegal; *; b * Public Hospitals of Paris, Dentistry Service, Pitié Salpêtrière University Hospital, Paris ,France*

**Keywords:** Canal Straightening, ProTaper, Reciproc, Root Canal Preparation, Root Canal Transportation, Rotary Instruments, WaveOne

## Abstract

**Introduction: **The aim of this study was to compare the shaping ability of two single-file systems and conventional rotary instruments in severely curved root canals of extracted human molars. **Methods and Materials: **Mesiobuccal canals of 120 mandibular molars with angles of curvature ranging between 25^°^ and 35^°^ and radii of curvature from 5 to 9 mm, were divided into three groups (*n*=40). In each group the canals were instrumented with either WaveOne (W), Reciproc (R) or ProTaper (P). The time required for canal shaping and the frequency of broken instruments were recorded. The standardized pre and post-instrumentation radiographs were taken to determine changes in working length (WL) and straightening of canal curvature. The presence of blockage or perforation was also evaluated. Data were analyzed using the one-way analysis of variance (ANOVA) and post-hoc Tukey’s test. The level of significance was set at 0.05. **Results: **Both single-instrument systems reduced the canal preparation time by approximately 50% (*P*<0.05). No incidence of broken instruments from single-file systems was reported; however, two F2 instruments in the P group were broken (*P*<0.05). Reduction in WL and straightening of canal curvature was observed in all three systems with the highest scores belonging to P system (*P*<0.05). No case of blockage or perforation was found during shaping in any group. **Conclusion**: Single-file systems shaped curved canals with substantial saving in time and a significant decrease in incidence of instrument separation, change in WL, and straightening of canal curvature.

## Introduction

Effective cleaning and shaping of the root canal system is essential to achieve the biological and mechanical objectives of endodontic treatment [1]. One of these objectives is the elimination of the organic content of the root canal system, as much as possible. In conjunction with this, gaining appropriate canal shaping will facilitate irrigation and three dimensional canal obturation [2, 3].

The use of engine driven rotary Nickel-Titanium (NiTi) instruments with continuous rotation helps the clinician achieve these objectives, but carries the risk of instrument separation and alternation of the original shape of the canal [4, 5]. Manufacturers have introduced different cross-sectional and longitudinal designs to minimize apical transportation and achieve faster and more predictable canal preparation [6]. In recent years, single-file systems have been introduced that meet the mechanical objectives of root canal cleaning and shaping [3, 7, 8]. 

Among these, Reciproc (VDW, Munich, Germany) has S-shaped cross-section, a non-cutting tip and sharp cutting edges that shapes the canal by means of a reciprocal back-and-forward motion with a speed of 300 rpm (150 degrees counterclockwise and then 30 degrees clockwise) [6]. This single file system is available at three different sizes and tapers; R25 (25/0.08), R40 (40/0.06) and R50 (50/0.05) [9].

**Figure 1.**
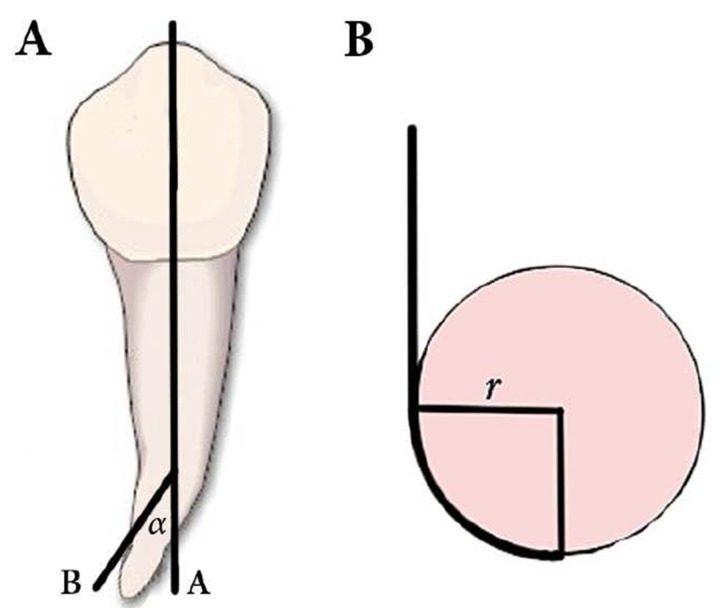
*A)* Measuring the angle of curvature (*α*): between lines *A* and *B*, *B) *Calculation of the radius (*r*) of a curved canal. The root canal is shown as a bold line.

WaveOne (Dentsply Maillefer, Ballaigues, Switzerland) is another single-file system. It has a reverse taper, variable helical angle and a non-active edge. It is used with 170^° ^counter clockwise rotation (direction of cutting) and 50^° ^clockwise rotations at a speed of 300 rpm. WaveOne is also available in different tip sizes and tapers; 20/0.06, 25/0.08 and 40/0.08 [10].

ProTaper (Dentsply Maillefer, Ballaigues, Switzerland) is amongst the pioneer engine driven instruments with full 360^°^ rotation. It has an active file design, with a convex triangular cross-section and an advanced flute design that has multiple tapers within the shaft. The basic system is comprised of three shaping (SX, S1 and S2) and three finishing (F1, F2, F3) instruments [11, 12].

The aim of the present study was to compare the shaping ability of WaveOne, Reciproc and ProTaper systems in severely curved mesiobuccal (MB) canals of extracted human mandibular molars. The null hypothesis was that there is no difference between these systems regarding preparation time and procedural accidents such as breaking of instruments, reduction of working length (WL), blockage, perforation and canal straightening.

## Materials and Methods

The study was conducted in the Conservative Dentistry and Endodontics service, Department of Odontology, Faculty of Medicine, Pharmacy and Odonto-Stomatology of the Cheikh Anta Diop University of Dakar.

A total of 120 extracted human mandibular molars with intact crowns and curved roots were selected. All molars were collected from three dental offices in Dakar, capital of Senegal. Extractions were indicated for periodontal or orthodontic reasons. The teeth showed no apical resorption or previous treatment. After debridement of the root surfaces, they were cleaned with soap and disinfected with 2.5% sodium hypochlorite (NaOCl). The teeth were then stored in saline solution for 15 days. Front and side preoperative anteroposterior radiographs were taken and an endodontic access cavity was prepared on each tooth. After navigation of the MB canals, teeth with apical canal diameter compatible with a #15 K-file (Dentsply Maillefer, Ballaigues, Switzerland), a radius of curvature ranging between 5.0 and 9.0 mm and an angle of curvature between 25˚ and 35˚ (according to Schneider’s method), were included [13]. Then a #15 K-file was placed in the MB canal and pre-instrumentation radiographies were taken. Radiographic images were amplified on a scale of 13×. Measurements were performed using Adobe Photoshop CS3 (Adobe Systems Inc., San Jose, CA). 

The angle of curvature was measured in both buccolingual and mesiodistal directions. A straight line (a) was plotted along the right coronary portion of the canal parallel to the long axis. The point where the canal deviates from this line to initiate the curvature was marked point A. A second line (b) was drawn to connect the apical foramen (point B) to point A. The angle of curvature was the acute angle formed by the straight lines (a) and (b) and was measured using Photoshop software (Figure 1A).

The curve between the points A and B is a circular arc, which defines the curved portion of the canal (Figures 1A and B). The radius of curvature can be calculated based on the measured length of the arc (S) between the points A and B. The arc (S) was measured using the computer program. The radius of curvature was calculated based on the geometrical principles of an isosceles triangle. The formula is as follows: R=S/2sin*α *where S is the arc between the points A and B and *α *is the angle of curvature. 

The initial WL was obtained by subtracting 1 mm from the length obtained after appearance of the tip of a K-file inserted in the canal. This length was verified using a radiography with front and side images. Based on the degree and the radius of curvature, the teeth were allocated into three identical groups of 40 teeth each. The homogeneity of the three groups with respect to the degree and the radius of curvature was assessed using analysis of variance (ANOVA) and post hoc Tukey’s test. 

During all of the radiography procedures, the teeth were placed in a tooth holder (Protrain, Simit Dental, Montova, Italy). The X-ray tube (Kodak, Paris, France) was oriented perpendicular to the axis of the roots. Radiographs of each root canal were taken from vestibular (clinical) view and mesial or distal (proximal) view. The exposure time was the same for all X-rays with a constant source-film distance of 50 cm and an object-film distance of 5 mm. Radiographic images were recorded in a computer and marked with a serial number. Root canal shaping of teeth was carried out, with each tooth maintained in tooth holder.

In group P, after evaluation of the initial WL, canal shaping was done with ProTaper instruments (Dentsply Maillefer, Ballaigues, Switzerland) in a brushing motion starting from S1 file, to reach the initial WL. Then S2, F1 and F2 were used to the WL according to the manufacturer’s instructions. The canals were irrigated with 2.5% NaOCl.

In groups W and R, after WL determination, the canals were shaped using the Primary WaveOne (25/0.08) or R25 Reciproc (25/0.08) files, respectively. The instruments were installed on a gear reduction handpiece (Sirona Dental Systems GmbH, Bensheim, Germany) powered by a torque-controlled motor (Silver; VDW GmbH, Munich, Germany) and were introduced into the canals with back-and-forth movements. After pecking for 2 or 3 times and when the blocking sensation was felt, the instrument was removed, cleaned and the canal was irrigated with NaOCl. This cycle was repeated until reaching the WL. All root canal shaping was carried out by a single senior operator.

In all groups the time required for complete and active shaping of the canal was recorded with a stop watch. The probable changes in the WL was determined by subtracting the final length (determined by taking a post-instrumentation radiography with a K-file) from the initial WL. The number of broken instruments during root canal shaping was also recorded. Blockages and perforations were also checked and documented.

The curvatures of the canals after instrumentation were redefined based on radiographies using the same initial technique [14]. The data were analyzed using the one-way analysis of variance (ANOVA) and post-hoc Tukey’s test and the level of significance was set at 0.05. 

## Results

The characteristics of the teeth are summarized in Table 1. The average time to shape a canal for each system showed that the single-instrumentation is much less time consuming compared to the ProTaper system (*P*<0.05). No instrument was broken in the W and R groups; whereas in the P group two F2 instruments were broken (*P<*0.05). Reductions in the WL were found with all systems (*P>*0.05). No case of blockage or perforation was found during the shaping with any of the three systems. However, canal straightening were noted for all groups (*P>*0.05).

## Discussion

This experimental laboratory study compared the root canal shaping and procedural accidents in MB canals of 120 mandibular molars by two single-instrument systems (Reciproc and WaveOne) and ProTaper instruments. Significant differences were found in terms of instrument fracture and duration of canal shaping in favor of single-file systems.

Two experimental models make it possible to study root canal shaping in laboratory: the resin simulator [15] or extracted teeth [1, 10, 16]. The use of resin simulators standardizes the diameter, length, angle and the radius of curvature of canals. Thus, variations in human teeth canals can be eliminated by using resin simulators. However, human teeth were used in this study for a better representation of clinical conditions. Indeed, resin is different from human dentine in terms of surface texture and hardness. The Knoop hardness value for the resin blocks is 36, whereas for dentine this value is between 40 and 72 according to Patterson [17]. In addition, the major drawback of simulators is the generation of heat caused by the friction of the rotating instruments, which can soften the resin that attaches to the blades of the instruments and cause their deformation or breakage. 

Despite the morphological variations in human teeth, several studies on root canal shaping between different systems have been carried out on extracted teeth [3, 18, 19]. The comparison of the three groups in this study showed good homogeneity between them. There were no statistically significant differences in the radius and angle of curvature between the three groups.

In this study, WaveOne and Reciproc files were compared to ProTaper files because these two systems of reciprocity are direct counterparts to ProTaper in its complete sequence [10]. Previous experimental studies have also evaluated the ProTaper instruments (F2) used in a reciprocal motion for the preparation of curved root canals [3, 18, 20]. To have similar apical shaping and diameter, the Reciproc R25, the primary WaveOne and the ProTaper F2 instruments were used because they all have similar tip sizes (#25).

Shaping with single-instrumentation takes less time compared to the ProTaper system. In a similar study comparing the shaping efficacy of Reciproc and WaveOne versus ProTaper, Bürklein *et al.* [10] described a significant 60% decrease in shaping time. This time saving with single-instrumentation systems is related to the simplicity of their use because a single instrument carries out the shaping unlike ProTaper, which requires the use of at least three instruments to obtain the same results. This can also have a technical explanation. Both WaveOne and Reciproc instruments have an inverted helix. The particular design of these instruments enables them to do the cutting action in counterclockwise direction more significantly than clockwise [21], thereby facilitating progression in an apical direction.

**Table 1:** Characteristics of teeth

**Systems**	**Degree of curvature (degrees)**			**Radius of curvature (mm)**		
	**Mean (SD)**	**Min**	**Max**	**Mean (SD)**	**Min**	**Max**
**ProTaper**	31.37 (3.30)	25.1	35	7.59 (1.13)	5.3	9
**WaveOne**	31.40 (3.28)	25	35	7.60 (1.08)	5.1	9
**Reciproc**	31.36 (3.27)	25.1	35	7.53 (1.11)	5.1	9
***P-value***	0.99			1.0		

No instruments from the Reciproc and WaveOne systems were broken. However, two broken instruments (F2) were reported for ProTaper system. This observation suggests that the single-instrument systems have a higher resistance to fracture. A study by Kim *et al.* [22] evaluated the resistance to fracture and cyclical fatigue of files used in reciprocal motion and reported similar results. This resistance to fracture can be attributed to the cross-sectional area of each instrument, which is higher for the WaveOne. Reciprocating instruments benefit from the qualities of the NiTi M-Wire alloy which is produced through a method of treatment of a NiTi wire by subjecting it to cycles of temperature change. This method increases the resistance to fracture [23, 24]. 

In addition, the reciprocating instruments have a variable angle and helical pitch to increase flexibility and permit better evacuation of debris but also a wider distribution of the blades than the ProTaper [22]. The results showed a decrease in the WL for the three systems used in this study. These results for NiTi instruments are consistent with those of other studies evaluating changes in WL using stainless steel files and Gates Glidden drills [25, 26]. Moreover, other studies focusing only on rotary NiTi instruments also showed a decrease in WL after root canal cleaning and shaping [16]. These decreases in WL are less significant with NiTi instruments compared to stainless steel files. This is probably due to the superior centering ability of NiTi instruments in the canal compared to stainless steel instruments. In this study, the largest reduction in WL was found with the ProTaper system. This finding is probably related to the important action of the S1 at the coronal portion of the canal. This action is particularly important if the curvature is notable. Indeed, in one study Berutti *et al*. [16] showed that two main factors influenced the change in WL after instrumentation with WaveOne and Reciproc. These factors are the root canal (curvature) and canal adjustment in the coronal region. In this study, the best results were obtained with the Reciproc system, which probably respects original canal anatomy.

No case of blockage or perforation was found during shaping with any of the systems. This finding is probably related to the methodology of the study. Indeed, initial patency significantly decreases the risk of modification of the original anatomy of the canal during shaping [15].

Concerning the straightening of canal curvature, the results obtained with the three systems showed mean values of angle of curvature between 2.37^˚^ and 3.16^˚^. These results are comparable to those of other studies carried out under similar experimental conditions [4, 5, 8, 18, 20]. Considering that the best results were obtained with the reciprocating instruments, it can be concluded that they are better at preparing curved canals respecting the original canal configuration [6, 27].

## Conclusion

The results of the present investigation confirm the superior ability of single-file systems to shape severely curved canals with less time and a significant decrease in procedural errors such as instrument fracture. However, no case of blockage or perforation was found during the shaping with any of the systems.
